# Systemic Primary Carnitine Deficiency Presenting With Substantia Nigra and Basal Ganglia Injury: A Case Report

**DOI:** 10.1002/jmd2.70014

**Published:** 2025-04-04

**Authors:** Tomoki Saito, Kento Soma, Mai Kashisaka, Kiiko Iketani, Masaaki Matsumoto, Takuya Ueda, Masahiro Nishiyama, Azusa Maruyama, Ryosuke Nakai, Hiroshi Sakihama, Hiroshi Kurosawa, Naoya Morisada, Hironori Kobayashi, Kayo Ozaki

**Affiliations:** ^1^ Department of Endocrinology and Metabolism Hyogo Prefectural Kobe Children's Hospital Kobe Japan; ^2^ Department of Neurology Hyogo Prefectural Kobe Children's Hospital Kobe Japan; ^3^ Department of Pediatric Critical Care Medicine Hyogo Prefectural Kobe Children's Hospital Kobe Japan; ^4^ Department of Clinical Genetics Hyogo Prefectural Kobe Children's Hospital Kobe Japan; ^5^ Laboratories Division Shimane University Hospital Izumo Japan

**Keywords:** dystonia, hypoketoic hypoglycemia, L‐DOPA, metabolic encephalopathy, systemic primary carnitine deficiency

## Abstract

Systemic primary carnitine deficiency (SPCD) is a rare congenital fatty acid metabolism disorder causing impaired β‐oxidation and energy production, leading to hypoglycemia, metabolic encephalopathy, and sudden death. Early diagnosis and treatment, including L‐carnitine supplementation and fasting avoidance, can improve prognosis. However, newborn screening (NBS) criteria differ by region, and standardized guidelines are lacking. This report presents a case of SPCD undetected by NBS, resulting in basal ganglia damage and dystonia due to metabolic decompensation. A 1‐year‐9‐month‐old girl with no abnormalities on NBS presented with impaired consciousness. She exhibited hypoketotic hypoglycemia, hyperammonemia, and myocardial hypertrophy. Suspecting a fatty acid metabolism disorder, L‐carnitine and high‐calorie infusion were initiated. Laboratory tests revealed markedly low serum total and free carnitine levels, and genetic analysis confirmed a homozygous *SLC22A5* mutation. Brain MRI on day 7 revealed bilateral basal ganglia and substantia nigra abnormalities. The patient developed severe dystonia and respiratory failure, requiring ECMO management. L‐DOPA was initiated on day 62, resulting in improvements in dystonia, swallowing, and motor function. By day 88, MRI showed resolution of basal ganglia abnormalities, though cerebral atrophy persisted. Basal ganglia damage is a rare but severe SPCD complication. L‐DOPA may alleviate dystonia by acting on dopaminergic neurons in the substantia nigra. Early ketone measurement during emergencies is crucial for diagnosing fatty acid metabolism disorders. A standardized NBS protocol with a defined carnitine cutoff value is essential for early detection and prevention of SPCD complications.

1

**Summary**

- Untreated systemic primary carnitine deficiency (SPCD) increases the risk of basal ganglia damage.
- Early diagnosis of SPCD through newborn screening (NBS) is critical for preventing severe complications.
- Establishing a standardized NBS protocol for the early detection of SPCD is desirable.

## 2 Introduction

Carnitine plays a critical role in energy production from fatty acids by transporting long‐chain fatty acids into the mitochondria, supplying substrates for β‐oxidation, and regulating the CoA/acyl‐CoA ratio within the mitochondria [1]. Systemic primary carnitine deficiency (SPCD) results from the dysfunction of the carnitine transporter (OCTN2) on the cell membrane, which reduces intracellular carnitine levels and impairs β‐oxidation and energy production [2]. During periods of increased energy demand (fever or exercise) or decreased supply (vomiting or fasting), severe hypoglycemia may occur due to the inability to utilize fatty acids. Organs dependent on β‐oxidation, such as the heart, skeletal muscles, liver, and brain (which has high energy demand but does not produce ketones), are particularly vulnerable [3, 4]. Early diagnosis often leads to a favorable prognosis and oral L‐carnitine supplementation, and the avoidance of fasting has been reported to be effective [3]. The measurement of blood‐free carnitine levels is included in newborn screening (NBS) programs in several countries to detect SPCD early [5, 6]. However, standardized criteria are lacking, and in some regions of Japan, formal guidelines were not established until recently.

Herein, we describe a case of SPCD that was not detected via NBS but was diagnosed in the emergency department. Despite promptly initiating therapeutic interventions for hypoketotic hypoglycemia, the patient developed substantia nigra and basal ganglia damage due to metabolic decompensation.

## 3 Case Report

A 1‐year and 9‐month‐old girl with mild motor developmental delay presented to the emergency department with altered consciousness. She was the first‐born child of nonconsanguineous parents with an uneventful perinatal history. Born at 41 weeks, she weighed 3060 g and showed no signs of distress. NBS revealed no abnormalities; however, the carnitine cutoff value in NBS yielded no suspicion of carnitine deficiency. She achieved head control at 3 months, transferred objects at 6 months, sat independently at 7 months, developed a pincer grasp at 10 months, pulled to stand at 14 months, and spoke multiple single words by 18 months.

Four days before the emergency department visit, she developed a fever > 40°C and nasal discharge; however, her oral intake remained adequate. On the morning of the presentation, she was lethargic and had skipped breakfast for the first time since she started eating solid foods. Nineteen hours after her last meal, she became unconscious and unresponsive to painful stimuli. She was transferred to our hospital after endotracheal intubation at the previous facility. During transit, normal saline was administered, but no glucose‐containing infusion was provided, as a glucose measurement had not been performed.

The patient presented with a height of 84.0 cm (−1.93 standard deviation (SD)), a weight of 10.0 kg (±0 SD), a temperature of 36.6°C, a heart rate of 130 bpm, a blood pressure of 97/32 mmHg, a respiratory rate of 20/min, and a SpO_2_ of 100% (PC‐SIMV mode, PEEP 10 H_2_O, ΔPS 14 H_2_O, FiO_2_ 0.5), with E1VTM1 consciousness level on the Glasgow Coma Scale. Head, neck, and chest examinations revealed no significant abnormalities. Abdominal examination revealed palpation of the liver 3 cm below the costal margin. No rashes were observed on the trunk or limbs. Echocardiography showed an ejection fraction (EF) of 57% and a left ventricular end‐systolic wall thickness of 7.3 mm, with mild left ventricular hypertrophy.

Simultaneously with glucose measurement, ketone levels were assessed using a rapid point‐of‐care device due to the patient's impaired consciousness and deterioration, raising suspicion of a congenital metabolic disorder. Consequently, hypoketotic hypoglycemia was confirmed with a blood < 20 mg/dL glucose level and a 3‐hydroxybutyrate level of 800 μmol/L. Rapid diagnosis of hypoketotic hypoglycemia allowed for the prompt initiation of treatment for metabolic dysfunction.

Table 1 presents the findings of the blood test obtained within 5 min of the intravenous injection of 40 mL of 20% glucose solution. Hypoketonemia (3‐hydroxybutyric acid: 485 μmol/L), marked hyperammonemia (> 500 μg/dL), and elevated levels of AST, ALT, and CK (155, 37, and 717 IU/L, respectively) were observed. Insulin level was < 0.30 μIU/mL, with no evidence of endocrine dysfunction. Furthermore, blood amino acid and urine organic acid analyses revealed no notable abnormalities. Acylcarnitines were all low, as shown in Table S1. Computed tomography (CT) of the head did not reveal any intracranial hemorrhage or edema. Electroencephalography revealed diffuse delta activity. Cardiac and abdominal ultrasonography showed left ventricular wall hypertrophy and hepatomegaly.

**TABLE 1:** Laboratory findings upon admission.

		Reference ranges	Units			Reference ranges	Units
Blood count				Biochemistry			
WBC	33	(43–191)	×10^2^/μL	Alb	3.1	(3.4–4.7)	g/dL
Hb	8.9	(10.7–14.1)	g/dL	T‐Bil	0.42	(0.2–0.7)	mg/dL
Plt	23.8	(16.8–65)	×10^4^/μL	AST	155	(24–57)	IU/L
				ALT	37	(9–38)	IU/L
Coagulation				LDH	544	(202–437)	IU/L
PT‐INR	1.18	(0.85–1.15)		CK	717	(39–295)	IU/L
APTT	33.7	(25–40)	s	UN	33.2	(3.7–18.6)	mg/dL
Fib	279	(150–400)	mg/dL	Cre	0.44	(0.16–0.32)	mg/dL
D‐dimer	5.1	(< 1.0)	μg/mL	CRP	0.41	(0–0.14)	mg/dL
				NH_3_	> 500	(35–100)	μg/dL
Arterial blood gas				Na	136	(135–143)	mmol/L
pH	7.102	(7.350–7.450)		K	4.2	(3.6–5.1)	mmol/L
pCO_2_	54.9	(35–45)	mmHg	Cl	109	(101–110)	mmol/L
HC$O_{3}^{-}$	16.4	(22.0–26.0)	mEq/L	Glu	288	(73–109)	mg/dL
BE	−12.9	(−2.0 to 2.0)	mEq/L	Lactate	13.7	(3–17)	mg/dL
				Pyruvate	0.9	(0.3–0.94)	mg/dL
Amino acid profile				GH	11.2	(0.13–9.88)	ng/mL
Valine	157.1	(–360)	nmol/mL	ACTH	187	(7.2–63.3)	pg/mL
Leucine + isoleucine	187.56	(–360)	nmol/mL	Cortisol	99	(7.07–19.6)	μg/dL
Methionine	17.64	(–60)	nmol/mL	Insulin	< 0.30	(1.84–12.2)	μIU/mL
Citrulline	14.39	(8–85)	nmol/mL	TSH	0.04	(0.38–5.38)	μIU/mL
Phenylalanine	86.46	(–150)	nmol/mL	FT_4_	0.67	(0.7–1.48)	ng/mL
Tyrosine	48.72	(–570)	nmol/mL	Free fatty acids	2058	(140–850)	μEq/L
Aruginine	1.94	(0.5–90)	nmol/mL	Acetoacetic acid	640	(–55)	μmol/L
Alanine	295.42	(N/A)	nmol/mL	3‐hydroxybutyric acid	485	(–85)	μmol/L
Orot acid	0.66	(–2.7)	nmol/mL				

Abbreviations: ACTH, adrenocorticotropic hormone; FT4, free thyroxine; GH, growth hormone; TSH, thyroid‐stimulating hormone.

**FIGURE 1:**
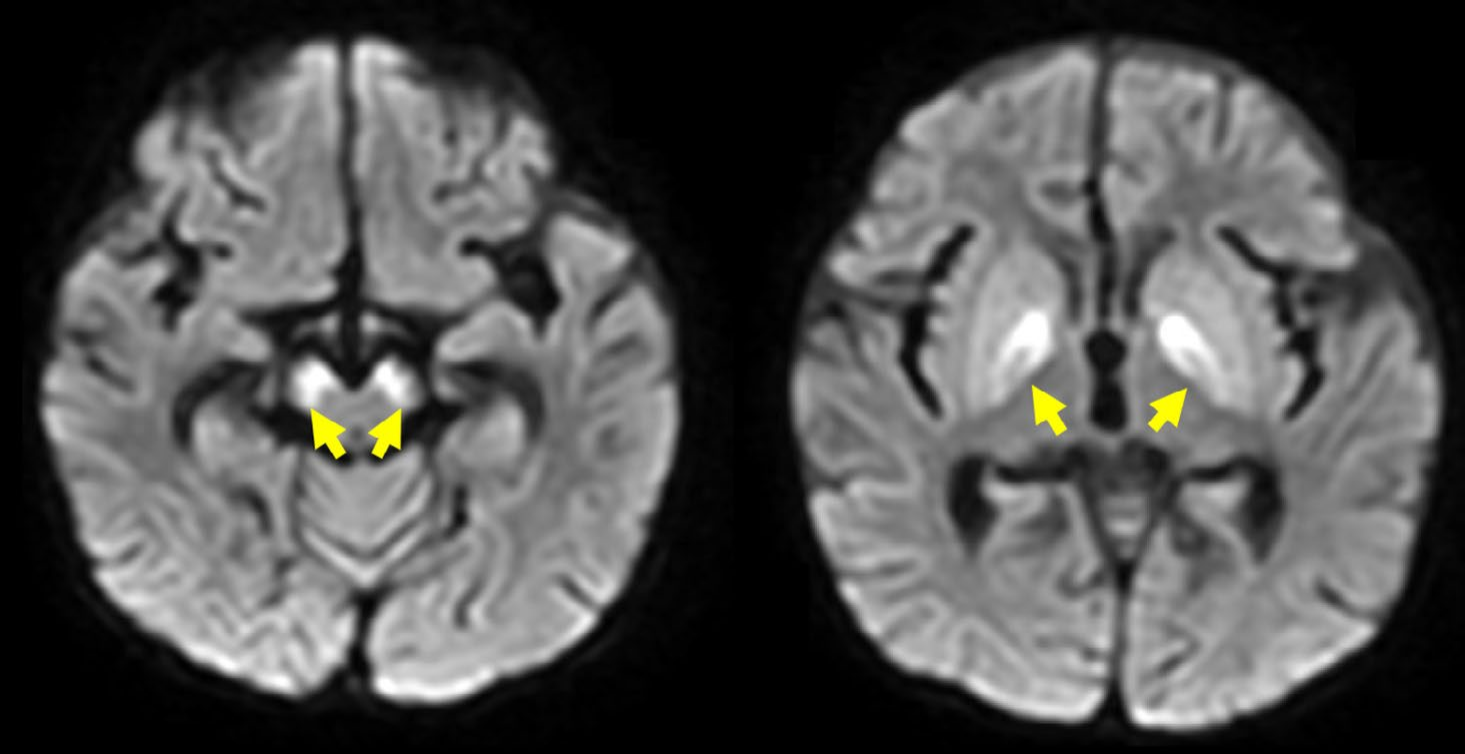
MRI on Day 7 after Admission. MRI showing abnormal signals in the bilateral globus pallidus and substantia nigra on diffusion‐weighted imaging (yellow arrows).

**FIGURE 2:**
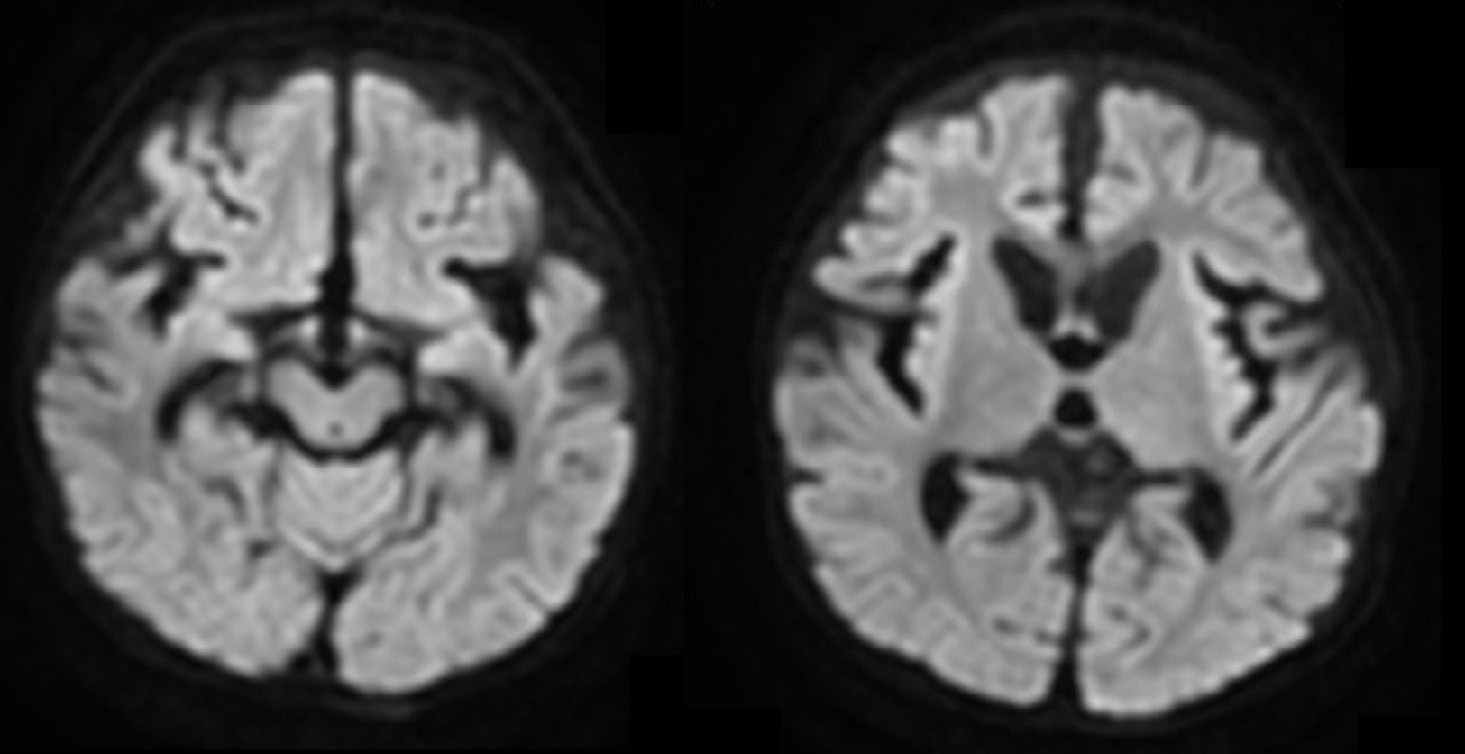
MRI on Day 88 after Admission. Cerebral parenchymal atrophy was observed on day 88 post‐admission. The abnormal signals observed in the bilateral globus pallidus and substantia nigra on diffusion‐weighted imaging have improved.

The patient was admitted to the intensive care unit, where mechanical ventilation was continued, and a high‐dose midazolam infusion was administered. Hyperammonemia was managed with sodium benzoate and arginine. Suspecting a metabolic disorder, a high‐calorie infusion (60 kcal/kg/day) was initiated, along with L‐carnitine (100 mg/kg/day) and a combination of vitamins (thiamine, riboflavin, pyridoxine, pantothenic acid, biotin, cobalamin, ascorbic acid, tocopherol, and ubiquinone). Cefotaxime was administered for suspected sepsis. By the third day post‐admission, blood ammonia levels had normalized, and treatment with sodium benzoate and arginine was discontinued. Blood cultures obtained at admission revealed the presence of *Streptococcus pneumoniae* . On the fifth day after admission, additional laboratory test findings revealed a considerably low serum total carnitine (1.56 μmol/L, reference 45**–**91 μmol/L) and serum‐free carnitine levels (0.53 μmol/L, reference 20**–**60 μmol/L) and a high urinary excretion rate of free carnitine (61.0%) These results suggested SPCD. Dried blood spots collected at birth were examined, revealing 2.137 μmol/L C0 (reference 20**–**60 μmol/L), 0.4 μmol/L C3 (reference—3.5 μmol/L), 0.7 μmol/L C16 (reference 0.5**–**3.0 μmol/L), and 0.119 C14/C3 (reference—0.4). Genetic testing identified a homozygous variant, c.506G>A (p.Arg169Gln), in the *SLC22A5* gene (NM_003060.4), which encodes the OCTN2 transporter, confirming the diagnosis of SPCD [7]. Head magnetic resonance imaging (MRI) performed on the seventh day after admission revealed symmetrical abnormal hyperintense signals in the bilateral globus pallidus and substantia nigra on diffusion‐weighted imaging (Figure 1). The patient was extubated on the eighth day after admission. Oral eperisone was initiated on the eleventh day after admission, as substantial muscle rigidity was noted. The patient was transferred to a general ward on the fourteenth day after admission. By day 26, the patient developed acute respiratory distress syndrome because of recurrent choking and aspiration owing to muscle rigidity and swallowing dysfunction, necessitating reintubation and extracorporeal membrane oxygenation (ECMO) management in the intensive care unit. The patient was weaned off ECMO on the 34th day after admission, and clonazepam was administered for muscle relaxation. The patient was extubated on the 46th day after admission; however, respiratory instability due to muscle rigidity persisted. Dantrolene was administered, and the patient required continued management in the intensive care unit because of recurrent choking. With generalized dystonia and MRI evidence of basal ganglia, including the substantia nigra and thalamic involvement, L‐3,4‐dioxyphenylalanine (L‐DOPA) was initiated at 1 mg/kg/day on the 60‐s day of hospitalization and gradually increased. Following L‐DOPA initiation, mandibular dystonia resolved immediately. By day 81, swallowing function had improved, allowing the patient to consume oral foods such as cotton candy and ice cream. Additionally, agitation and respiratory instability improved, leading to the discontinuation of eperisone and dantrolene on the 80‐s day. By day 88, brain MRI revealed the resolution of high signal intensity in the basal ganglia on diffusion‐weighted imaging (DWI), although cerebral parenchymal atrophy was observed (Figure 2). By day 98, the L‐DOPA dosage was increased to 5 mg/kg/day, administered in five divided doses per day. The Fahn–Marsden scale, a widely used tool for dystonia severity assessment by quantifying movement scores, improved from 81.5/120 before L‐DOPA administration to 28.5/120 after the dose increase to 5 mg/kg/day. By day 118, echocardiography showed an ejection fraction (EF) of 66% and a left ventricular end‐systolic wall thickness of 6.7 mm, indicating improvements in cardiac contractility and left ventricular wall hypertrophy. Oral L‐carnitine at 200 mg/kg/day stabilized blood‐free carnitine levels, and the patient was discharged on day 121.

Following the patient's discharge, the birth of her younger sister was scheduled for 2 months later. Genetic counseling informed the parents of a 25% risk of SPCD in the second child. Hospital conferences discussed this risk, stressing hypoglycemia prevention. However, metabolic decompensation was deemed unlikely if breastfeeding frequency and quantity were adequate. The sister was born vaginally, underwent postnatal glucose monitoring and fluid management, and showed a 12.14 μmol/L free carnitine level in NBS. Genetic testing was performed after birth, but no *SLC22A5* mutations were found.

## 4 Discussion

Basal ganglia damage is a rare complication of SPCD. Khries et al. reported a case of an 8‐year‐old boy who presented with fever and impaired consciousness, showing high signal intensity in the lentiform and caudate nuclei on fluid‐attenuated inversion recovery (FLAIR) imaging [8]. Yilmaz et al. described a 12‐year‐old boy who exhibited prolonged impaired consciousness and tremors lasting more than a week, with MRI revealing symmetrical high signal intensity on diffusion‐weighted imaging (DWI) in the corona radiata, deep cerebellar white matter, and ascending and descending brainstem tracts [9]. Untreated carnitine deficiency increases the risk of metabolic encephalopathy due to hypoglycemia and mitochondrial dysfunction. Reports of SPCD presenting with basal ganglia damage, including the substantia nigra, as in this case, are rare and exhibit imaging findings distinct from those in previous literature. In this case, motor impairment due to damage to the basal ganglia was observed as a sequela, with dystonia being especially prominent. L‐DOPA is the first‐line treatment for dopa‐responsive dystonia. It has been demonstrated to improve abnormal movements, posture, and gait in patients with dopa‐responsive dystonia; however, the response may be variable or delayed [10]. As a dopamine precursor, L‐DOPA crosses the blood–brain barrier and is converted into dopamine in the brain, thereby exerting its effects. It is often combined with a peripheral dopa‐decarboxylase inhibitor such as carbidopa. However, carbidopa is not indicated for pediatric patients in Japan. Therefore, L‐DOPA was used alone, with gradual dosage titration and frequent administration, to achieve clinical effectiveness. The substantia nigra contains numerous dopaminergic neurons [11]. In this case, imaging findings suggested dysfunction of the globus pallidus and substantia nigra, making L‐DOPA treatment a rational choice. The patient exhibited severe respiratory and swallowing difficulties due to dystonia, which were challenging to manage with eperisone, dantrolene, and benzodiazepines. However, dystonia improved following the administration of L‐DOPA. This suggests that L‐DOPA could be considered a potential treatment for dystonia due to basal ganglia injury following metabolic decompensation. However, further case accumulation is necessary to establish its efficacy.

In the present case, early diagnosis of hypoketotic hypoglycemia using a rapid measuring device in the emergency department enabled prompt recognition of fatty acid metabolism disorders, facilitating swift treatment initiation. Although most cases of hypoglycemia encountered in the emergency setting are idiopathic, endocrine disorders and metabolic abnormalities should always be considered in the differential diagnosis. Measuring ketone bodies is a crucial first step in the diagnostic process, as non‐ketotic hypoglycemia suggests either hyperinsulinemic hypoglycemia or a β‐oxidation disorder [12]. The acylcarnitine profile and urine organic acid analysis are also important in the initial evaluation, as they not only support the diagnosis of SPCD but also help exclude other metabolic abnormalities that may result in secondary carnitine deficiency. In this case, SPCD was confirmed based on these test findings and the identification of a known disease‐associated mutation through genetic analysis [7]. The patient's motor developmental delay may have been caused by muscle weakness associated with SPCD, whereas hyperammonemia was likely due to mitochondrial dysfunction resulting from carnitine deficiency.

The measurement of blood‐free carnitine concentration is incorporated into NBS programs in several countries to facilitate the early detection of SPCD. However, the criteria differ between nations [13]. NBS using free carnitine as an indicator can be affected by factors such as prematurity, parenteral nutrition management, maternal carnitine deficiency, and nutritional status at the time of blood collection. Additionally, they may be influenced by secondary carnitine deficiency resulting from inborn metabolic disorders such as glutaric acidemia, methylmalonic acidemia, and MCAD deficiency [14, 15]. Notably, false‐positive results are common, with a positive predictive value ranging from approximately 4% to 20% [5, 6]. The prevalence of SPCD in Japan is estimated to be approximately one in 40 000 individuals [16]. However, reports have indicated detection frequencies as low as one in 260 000 individuals [17]. Currently, no reliable method exists for the early and accurate diagnosis of SPCD, although algorithms that include additional biomarkers alongside free carnitine are being developed [13]. The municipality where the patient was born established a cutoff value for free carnitine concentration (< 6.6 μmol/L) in 2021, 1 month after the patient's birth. Had this criterion been in place at the time of the patient's birth, the case might not have been overlooked. Although free carnitine concentration was measured during the newborn metabolic screening test at the time of the patient's birth, and a low level was recognized, no specific actions were taken due to the absence of an established cutoff value. This case highlights the importance of NBS and underscores the necessity of sharing abnormal findings with parents and healthcare providers. Furthermore, it emphasizes the need for a nationally standardized and clearly defined cutoff value for free carnitine concentration to improve the early detection of SPCD.

## 5 Conclusion

Basal ganglia damage, including involvement of the substantia nigra, is a rare complication of SPCD, and L‐DOPA may be effective in treating dystonia. Hypoketotic hypoglycemia is indicative of fatty acid metabolism disorders and can be promptly recognized even in the emergency department using rapid measuring devices. Although a standardized method for NBS has not yet been established, early diagnosis of SPCD can significantly improve prognosis.

## Author Contributions

Tomoki Saito drafted the primary manuscript. Tomoki Saito, Kento Soma, and Kayo Ozaki conceptualized the study. Naoya Morisada and Hironori Kobayashi provided expertise in biochemical and laboratory interpretations. Takuya Ueda, Masahiro Nishiyama, Azusa Maruyama, Ryosuke Nakai, Hiroshi Sakihama, and Hiroshi Kurosawa contributed to the interpretation of physical findings and imaging evaluations with their expertise. Mai Kashisaka, Kiiko Iketani, and Masaaki Matsumoto contributed to patient care and manuscript editing, providing valuable insights into the treatment of metabolic disorders. All authors reviewed the manuscript and approved the final version for publication.

## Ethics Statement

The authors have nothing to report.

## Consent

All procedures followed the ethical standards of the responsible committee on human experimentation (institutional and national) and with the Helsinki Declaration of 1975, as revised in 2000 (5). Informed consent was obtained from the patient's parents for inclusion in the study. In addition, written informed consent was obtained from the patient's parents to publish anonymized information in this article.

## Conflicts of Interest

The authors declare no conflicts of interest.

## Supporting information


**Supplementary Table S1.** Acylcarnitine profile.

## Data Availability

Anonymized data presented in this manuscript are available for review upon reasonable request.
